# Supplementary material for: Extracts from Environmental Strains of Pseudomonas spp. Effectively Control Fungal Plant Diseases
Source: Plants (Basel). 2022 Feb 5;11(3):436. doi: 10.3390/plants11030436 (PMC8840005; doi:10.3390/plants11030436)
Supplement: Supplementary file 1 [file plants-11-00436-s001.zip › plants-1582685-supplementary.pdf]

Supplementary material

Supplementary figures

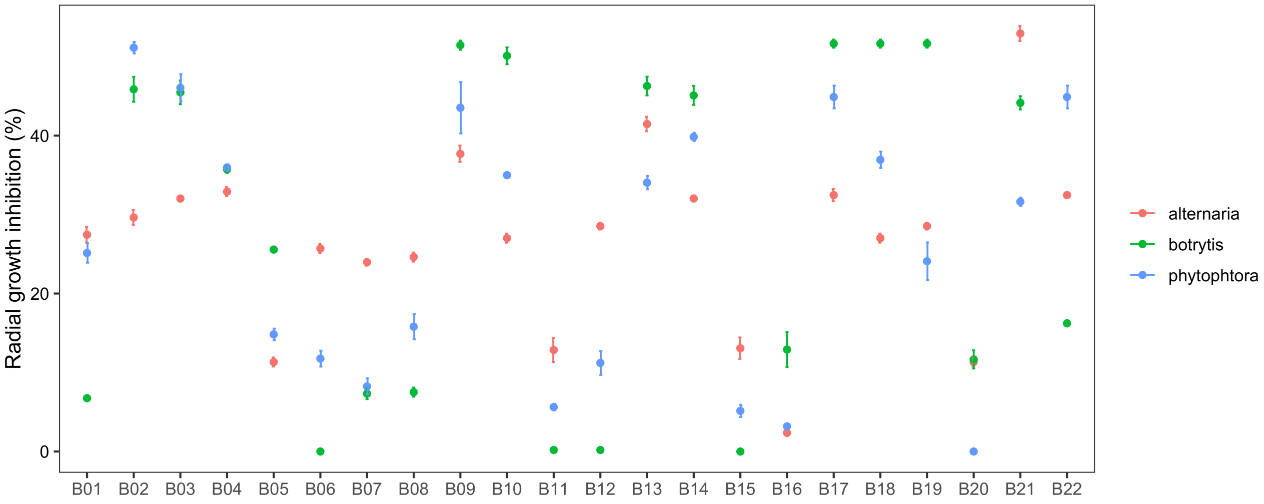

Figure S1: Inhibition of fungal growth by each microbial isolate in the dual-culture assay, for each isolate-pathogen combination.

Supplementary tables

**Locality**	**Prevalent plant species**	**GPS coordinates**	**Collected bacteria (N.)**	**Selected bacteria isolates**
Reggio Calabria	*Pinus pinea*	38°07'10''N/15°40'05''E	17	B02, B03, B09, B17, B18
		38°07'12''N/15°40'01''E	23	B05, B15, B10
		38°07'16.3''N/15°40'07.1''E	18	B13, B16, B19, B20
Serra S. Bruno	*Abies alba*	38°33'42.4"N/16°18'54.2"E	20	B01, B11, B12
Stalettì	*Quercus ilex*	38°45'26.7"N/16°33'16.4"N	15	B04
Arena	*Fagus sylvatica*	38°33'43.1"N/16°12'37.4"E	19	B06, B07, B08, B14, B22
Bova	*Pinus nigra laricio*	38°02'15.9"N/15°57'14.1"E	13	B21
**Total**	~	~	**125**	**22**

Table S1: Details about the sampling sites, the number of bacteria isolated, and those that were selected to be tested in this study.

Isolate	Tomato - *Botrytis*		Tomato - *Alternaria*		Apple - *Penicillium*		Grape - *Botrytis*		Olive - *Colletotrichum*		Tangerine - *Penicillium*		Tot
	Chisq	*p*	*Chisq*	*p*	*Chisq*	*p*	*Chisq*	*p*	*Chisq*	*p*	*Chisq*	*p*	
**B01**	2.91	0.08	9.83	**<0.001**	0.00	1	25.89	**<0.001**	4.31	**0.03**	12.32	**<0.001**	**4**
**B02**	4.49	**0.03**	7.93	**<0.001**	0.00	1	17.73	**<0.001**	7.38	**0.006**	19.66	**<0.001**	**5**
**B03**	4.49	**0.03**	0.00	1	0.00	1	19.66	**<0.001**	7.38	**0.006**	8.98	**0.002**	**4**
**B04**	1.42	0.23	1.42	0.23	0.00	1	8.98	**0.002**	10.63	**0.001**	4.31	**0.03**	**3**
**B05**	34.11	**<0.001**	4.49	**0.03**	34.11	**<0.001**	44.06	**<0.001**	74.34	**<0.001**	63.07	**<0.001**	**6**
**B06**	4.49	**0.03**	1.42	0.23	0.00	1	4.31	0.03	58.54	**<0.001**	12.32	**<0.001**	**4**
**B07**	7.93	**<0.01**	0.00	1	0.00	1	2.84	0.09	54.46	**<0.001**	2.84	0.09	**2**
**B08**	6.16	**0.01**	1.42	0.23	0.00	1	32.92	**<0.001**	74.43	**<0.001**	1.41	0.2	**3**
**B09**	29.2	**<0.001**	7.93	**<0.001**	41.58	**<0.01**	58.54	**<0.001**	58.54	**<0.001**	74.34	**<0.001**	**6**
**B10**	0.00	1	0.00	1	0.00	1	83.17	**<0.001**	58.54	**<0.001**	12.32	**<0.001**	**3**
**B11**	0.00	1	1.42	0.23	0.00	1	0	1	68.21	**<0.001**	0	1	**1**
**B12**	0.00	1	0.00	1	0.00	1	0	1	0	1	0	1	0
**B13**	0.00	1	0.00	1	0.00	1	0	1	0	1	0	1	0
**B14**	1.42	0.23	0.00	1	0.00	1	0	1	0	1	0	1	0
**B15**	0.00	1	0.00	1	0.00	1	0	1	0	1	0	1	0
**B16**	1.42	0.23	0.00	1	0.00	1	0	1	0	1	0	1	0
**B17**	0.00	1	0.00	1	0.00	1	0	1	0	1	0	1	0
**B18**	1.42	0.23	0.00	1	0.00	1	0	1	0	1	0	1	0
**B19**	0.00	1	0.00	1	0.00	1	0	1	0	1	0	1	0
**B20**	0.00	1	0.00	1	0.00	1	0	1	0	1	0	1	0
**B21**	1.42	0.23	0.00	1	0.00	1	0	1	0	1	0	1	0
**B22**	0.00	1	0.00	1	0.00	1	0	1	0	1	0	1	0

Table S2: Results from testing the decay incidence in fruits treated with each bacterial isolates against the negative control (always 100% of fruits showing rots), for each host-pathogen combination. Values in bold indicate a significant reduction of disease incidence in fruits treated with the isolate and those in the control group. The last column indicates the number of trials in which each isolate successfully controlled the development of the fungal pathogen.

Supplementary results

Molecular identification of selected isolates

We identified the three bacterial isolates that resulted more effective during field trials (B01, B05, B09) by sequencing a portion of the 16 rRNA. DNA was extracted from bacterial cells collected from single colonies using a sterile loop and suspended in 300 µl of sterile ultrapure water in a 1.5 ml tube. After brief vortexing, bacterial suspensions were mechanically lysed in a Ribolyzer for 30 s at 6.5 Hz. Tubes were centrifuged for 3 min at 1200 x g, and the supernatant was then transferred into a clean tube and directly used for PCR amplifications. The 16S region was amplified with primers 27F-1492R (Heuer et al., 1997). Amplifications were performed in a volume of 30 µL containing ∼100 ng of template DNA, 10 µM of each primer and 6 µl of Taq&Go polymerase (MP Biomedicals). PCR amplification conditions consisted of 1 cycle at 94°C for 4 min; 25 cycles at 98°C for 30 s, 50°C for 30s, 72 °C for 90s, and a final cycle at 72°C for 5 min. PCR products were purified with the MinElute PCR Purification Kit (Quiagen Ltd.West Sussex, UK), prepared using a dye-terminator cycle-sequencing reaction (FS sequencing kit, Applied Biosystems,Warrington, UK) and sequenced on an ABI373 automated sequencer (Applied Biosystems). Sequences were then manually curated using the software CHROMASPRO v. 1.5 (http://www.technelysium.com.au/). Sequences were compared with those deposited in GenBank using BLASTn, which suggested that all three isolates belong to the genus Pseudomonas. To further improve this identification, we compared our sequences with those available on SILVA (Quast et al., 2012), by aligning sequences using MUSCLE (Edgar, 2004), trimming them to the same length, and building a phylogenetic tree using the Maximum Likelihood method and the Tamura-Nei models as implemented in MEGA X (Kumar et al., 2018), bootstrapped 500 times (Fig. S2).

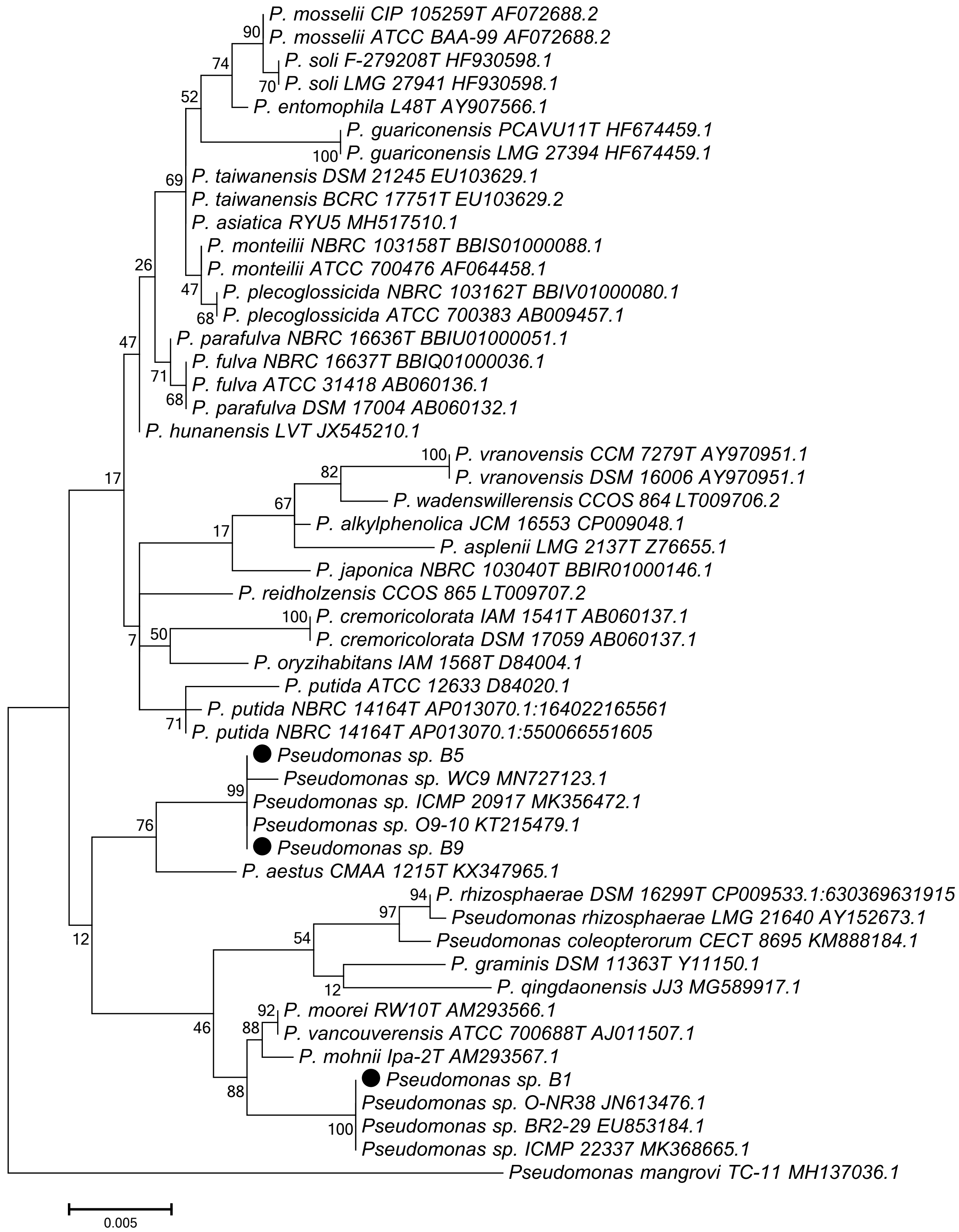

Figure S2: Phylogenetic tree comparing the 16S rRNA of isolates B01, B05, and B09 with a collection of sequences available on SILVA (Quast et al., 2012).

References

Edgar, R. C. (2004). MUSCLE: multiple sequence alignment with high accuracy and high throughput. *Nucleic acids research*, 32(5):1792--1797.

Heuer, H., Krsek, M., Baker, P., Smalla, K., and Wellington, E. (1997). Analysis of actinomycete communities by specific amplification of genes encoding 16S rRNA and gel-electrophoretic separation in denaturing gradients. *Applied and environmental microbiology*, 63(8):3233--3241.

Kumar, S., Stecher, G., Li, M., Knyaz, C., and Tamura, K. (2018). MEGA X: molecular evolutionary genetics analysis across computing platforms. *Molecular biology and evolution*, 35(6):1547.

Quast, C., Pruesse, E., Yilmaz, P., Gerken, J., Schweer, T., Yarza, P., Peplies, J., and Glöckner, F. O. (2012). The SILVA ribosomal RNA gene database project: improved data processing and web-based tools. *Nucleic acids research*, 41(D1):D590--D596.
